# The interaction among OSA, CPAP, and medications in patients with comorbid OSA and cardiovascular/cerebrovascular disease: a randomized controlled trial

**DOI:** 10.1186/s12890-022-01879-2

**Published:** 2022-03-21

**Authors:** Miaochan Lao, Yilu Cheng, Xinglin Gao, Qiong Ou

**Affiliations:** 1 Department of Sleep Center, Department of Geriatric Respiratory, Guangdong Provincial People’s Hospital, Guangdong Academy of Medical Sciences, Guangdong Provincial Geriatrics Institute, No. 106 Zhongshan Er Rd, Guangzhou, 510080 China; 2 Geriatric Respiratory Department I, Guangdong Provincial People’s Hospital, Guangdong Academy of Medical Sciences, Guangdong Provincial Geriatrics Institute, Guangzhou, China

**Keywords:** Obstructive sleep apnea, Cardiovascular disease, Cerebrovascular disease, Medication, Prognosis

## Abstract

**Background:**

Most patients with comorbid sleep apnea (OSA), cardiovascular (CV) disease, and/or cerebrovascular (CeV) disease simultaneously take medications. Whether OSA and continuous positive airway pressure (CPAP) interact with CV/CeV medications remains unknown. This study aimed to determine the interaction among OSA, CPAP, and CV/CeV medications; the effects of medications on major adverse cardiac and cerebrovascular events, and survival in patients with comorbid OSA and CV/CeV.

**Methods:**

This was a post hoc analysis of the data from one center of the Sleep Apnea Cardiovascular Endpoints Study. Participants (aged 45–75 years) with comorbid OSA and CV/CeV were randomized to receive usual care with or without CPAP from December 2008 to November 2013. The primary endpoint was death and the secondary endpoint was a composite of death, myocardial infarction, stroke, hospitalization for unstable angina, heart failure, and transient ischemic attack.

**Results:**

In total, 131 patients were analyzed. Sixty-three were in the CPAP group and 68 were in the usual care group, 41 had good adherence to CPAP (65.1%), and the median follow-up time was 43.0 (35.0, 54.0) months. In Cox regression analysis, ACE inhibitors and nitrates were independent factors for decreased survival in patients with comorbid OSA and CV/CeV (chi-square = 22.932, *P* = 0.003; ACE inhibitors: OR 7.241, *P* = 0.048, 95% CI 1.016–51.628; nitrates: OR 18.012, *P* = 0.011, 95% CI 1.923–168.750). ACE inhibitors increased mortality and secondary endpoints in the CPAP group (chi-square = 4.134, *P* = 0.042) but not in patients with good CPAP adherence. Clopidogrel and nitrates decreased survival in usual care group (clopidogrel: chi-square = 5.312, *P* = 0.021; nitrates: chi-square = 6.417, *P* = 0.011), but not in CPAP group.

**Conclusions:**

OSA may predispose patients with CV/CeV and CV/CeV medications to a negative effect. CPAP treatment may neutralize the negative effects of OSA by relieving chronic intermittent hypoxia.

*Trial registration* ClinicalTrials.gov (NCT00738179, first registration date: 20/08/2008).

## Background

Obstructive sleep apnea (OSA) is a common complication in patients with cardiovascular (CV) and cerebrovascular (CeV) diseases. OSA was found in 45.3% of patients who underwent percutaneous coronary intervention [1] and 38–72% of patients with ischemic, hemorrhagic stroke, and transient ischemic attack (TIA) [2]. The recurrence of major adverse cardiac and cerebrovascular events (MACCEs) was as high as 15.4–18.9% after a mean follow-up of 3–3.7 years in patients with comorbid OSA and CV/CeV [1, 3]. The Sleep Apnea Cardiovascular Endpoints (SAVE) Study demonstrated that continuous positive airway pressure (CPAP), as a first-line treatment for OSA, could not prevent cardiovascular events in patients with OSA and established CV/CeV [3]. Current guidelines for the management of patients with OSA and CV/CeV demonstrate the impact of OSA on the prognosis of CV/CeV [4, 5]. However, these guidelines do not illustrate the impact of OSA on the effects of CV/CeV medications. How CV/CeV medications interact with OSA and CPAP, and their impact on the recurrence of MACCEs and survival are unknown. To make sure the effect of a specific medication on patients with OSA and CV/CeV is important for clinical practice. The current study aimed to determine the interaction among OSA, CPAP, and CV/CeV medications, the effects of medications on MACCEs, and survival in patients with comorbid OSA and CV/CeV.

## Methods

### Participants and study design

This was a post hoc analysis of the data from one center of the SAVE study (an international multicenter, randomized controlled trial) that evaluated the effectiveness of CPAP in reducing recurrent cardiovascular events and cardiovascular-related deaths in patients with comorbid OSA and cardiovascular or cerebrovascular disease [3]. Detailed inclusion and exclusion criteria are listed in the Supplementary Information of the SAVE report [3]. Briefly, the inclusion criteria were an age between 45 and 75 years; a diagnosis of coronary artery disease or cerebrovascular disease; and a diagnosis of moderate-to-severe OSA. Evidence of coronary artery disease was defined as previous myocardial infarction (MI), history of angina with documented coronary artery disease on angiography, exercise stress test, positive perfusion scintigram, or multi-vessel coronary revascularization, including coronary artery bypass graft (CABG) surgery or percutaneous transluminal coronary angioplasty (PTCA). Evidence of cerebrovascular disease was defined as previous stroke (including definite or presumed cerebral ischemia/infarction and intracerebral hemorrhage, but not subarachnoid hemorrhage), minor disabling stroke with minimal residual neurological disability, or a previous transient ischemic attack (TIA) of the brain or retina. A ≥ 4% arterial saturation oxygen dip rate of ≥ 12 per hour detected by a home sleep test (ApneaLink, ResMed, Munich) and confirmed centrally by sleep specialists was diagnosed as moderate-to-severe OSA.

The exclusion criteria included comorbid disease with severe disability or a high likelihood of death within the next 2 years; planned coronary or carotid revascularization procedure within the next 6 months; severe respiratory disease; the New York Heart Association classification class III–IV of heart failure; neurological deficits that prevented self-administration of the CPAP mask; a contraindication to the use of CPAP; prior use of CPAP treatment; other household members enrolled in the SAVE trial or using CPAP; increased risk of a sleep-related accident and/or excessive daytime sleepiness; severe nocturnal desaturation, defined as > 10% overnight recording time with nocturnal arterial oxygen saturation of < 80% documented on the ApneaLink device; Cheyne-Stokes respiration; residence remote from the clinic to preclude follow-up clinic visits.

A total of 151 participants (aged 45–75 years) with comorbid moderate-to-severe OSA and CV/CeV were randomized to receive usual care with or without CPAP at Guangdong Provincial People’s Hospital from December 2008 to November 2013. The study was approved by the ethics committee of Guangdong Provincial People’s Hospital and was performed in accordance with the ethical standards laid down in the 1964 Declaration of Helsinki and its later amendments. Written informed consent was obtained from all the participants. This study was registered at ClinicalTrials.gov (NCT00738179).

### Procedures and outcomes

At the time of enrolment, the demographic characteristics, medical history, and medications of the participants were recorded. Participants were assigned to receive usual care with or without CPAP treatment, after a 1-week run-in phase of sham CPAP and randomization. Follow-ups were conducted at 1, 3, 6 and 12 months, and 6 monthly thereafter. The primary endpoint of our study was death and the secondary endpoint was a composite of death, myocardial infarction, stroke, hospitalization for unstable angina, heart failure, and transient ischemic attack.

### Statistical analysis

Analyses were conducted using the SPSS software (version 25.0; IBM SPSS Statistics for Windows; Armonk, NY, USA). Normality tests were performed using the Shapiro–Wilk test. Normally distributed variables are presented as mean ± SD, and non-normally distributed variables are presented as median (25% and 75% interquartile range). Qualitative variables are presented as frequency and ratio of cases over the group. Comparisons between the CPAP group and usual care group were performed using the *t*-test for normally distributed variables, Wilcoxon Rank-Sum test for non-normally distributed variables, and chi-square test for qualitative variables. Chi-square test was used to estimate the relationship between medications and endpoints. Kaplan–Meier analysis was used to estimate the impact of sex, age (≥ 65 years), obesity (BMI ≥ 25 was defined as obesity), CPAP treatment, and medication on the primary and secondary endpoints. Sex, age, obesity, CPAP treatment, and medications were entered into Cox regression analysis to estimate the risk factors for the primary and secondary endpoints. The statistical significance level for all analyses was set at *P* = 0.05.

## Results

### Baseline characteristics

The baseline characteristics of the study participants are presented in Table 1. In total, 151 participants were enrolled in this study. 16 changed OSA treatment during follow-up, 14 dropped CPAP, and 2 initiated CPAP. Five changed the medication during follow-up, among which one changed the OSA treatment. Therefore, 131 patients were included in the analysis. 63 were in the CPAP group, and 68 were in the usual care group. The mean CPAP adherence was 4.4 ± 1.7 h per night (95% CI 3.9–4.8) in the CPAP group. Of the 63 patients in the CPAP group, 41 (65.1%) had good adherence to treatment (≥ 4 h per night). The median number of medications usage was 4.0 (3.0, 5.0). There were no significant differences in the number and types of medications between the CPAP and usual care groups. The median follow-up time was 43.0 (35.0, 54.0) months. There was no significant difference in the incidence rates of the primary and secondary endpoints between the CPAP and usual care groups.

**Table 1:** Baseline characteristics of the study participants

	CPAP group (N = 63)	Usual-care group (N = 68)	*P*
Age, years	62.2 ± 7.2	61.5 ± 7.4	0.563
Male, N (%)	53 (84.1%)	55 (80.9%)	0.626
Anthropometric measurements			
BMI, kg/m^2^	43 (68.3%)	47 (69.1%)	0.915
Waist circumference, cm	92.4 ± 8.7	92.9 ± 9.8	0.754
Hip circumference, cm	98.0 (94.0, 103.0)	101.0 (97.0, 105.0)	0.100
Neck circumference, cm	38.6 ± 3.2	38.6 ± 3.2	0.981
Waist-hip ratio	0.93 ± 0.05	0.92 ± 0.05	0.171
Systolic pressure, mmHg	120.0 (115.0, 130.0)	120.0 (110.0, 130.0)	0.427
Diastolic pressure, mmHg	70.0 (64.0, 80.0)	70.0 (65.8, 80.0)	0.745
Pulse, beats/min	63.0 (56.0, 68.0)	63.0 (59.0, 68.0)	0.452
Sleep monitoring			
AHI, per hour	23.0 (16.0, 35.5)	24.5 (17.5, 41.8)	0.547
ODI, per hour	19.5 (15.0, 35.5)	24.5 (16.0, 37.0)	0.410
Snoring, per hour	1499.0 (659.3, 2352.3)	1198.5 (634.0, 2430.8)	0.734
Mean oxygen saturation, %	94.0 (93.0, 95.0)	94.0 (93.0, 95.0)	0.673
Nidir oxygen, %	81.0 (72.0, 83.0)	78.0 (72.0, 82.0)	0.381
Time with oxygen saturation less than 90%, min	22.0 (8.8, 60.8)	24.0 (12.0, 55.5)	0.569
ESS	7.0 (5.0, 9.0)	5.0 (4.3, 6.0)	< 0.001
Sham CPAP, hours	5.4 ± 1.3	5.5 ± 1.2	0.654
Comorbidity			
Myocardial infarction, N (%)	21 (33.3%)	24 (35.3%)	0.813
PTCA, N (%)	26 (41.3%)	28 (41.2%)	0.991
CABG, N (%)	5 (7.9%)	0 (0.0%)	0.018
Angina, N (%)	20 (31.7%)	22 (32.4%)	0.941
Heart failure, N (%)	1 (1.6%)	2 (2.9%)	0.605
Hypertension, N (%)	55 (87.3%)	59 (86.8%)	0.927
Stroke, N (%)	27 (42.9%)	29 (42.6%)	0.981
TIA, N (%)	5 (7.9%)	4 (5.9%)	0.642
Diabetes mellitus, N (%)	20 (31.7%)	16 (23.5%)	0.293
Medications			
Aspirin, N (%)	39 (61.9%)	49 (72.1%)	0.216
Clopidogrel, N (%)	27 (42.9%)	34 (50.0%)	0.413
Statin, N (%)	42 (66.7%)	42 (61.8%)	0.559
Beta-blocker, N (%)	27 (42.9%)	33 (48.5%)	0.515
ARB, N (%)	24 (38.1%)	25 (36.8%)	0.875
ACE inhibitors, N (%)	9 (14.3%)	9 (13.2%)	0.861
CCB, N (%)	32 (50.8%)	31 (45.6%)	0.551
Nitrates, N (%)	2 (3.2%)	6 (8.8%)	0.177
Insulin, N (%)	0 (0.0%)	1 (1.5%)	0.334
Antidiabetic oral medication, N (%)	12 (19.0%)	13 (19.1%)	0.992
Chinese medicine, N (%)	2 (3.2%)	2 (2.9%)	0.938
Number of medication usage	4.0 (3.0, 5.0)	4.0 (3.0, 5.0)	0.540
Death, N (%)	3 (4.8%)	5 (7.4%)	0.536
Secondary endpoint, N (%)	8 (12.7%)	13 (19.1%)	0.317
Follow-up time, months	42.0 (32.0, 54.0)	43.5 (36.0, 54.8)	0.810

*CPAP* continues positive airway pressure, *BMI* body mass index, *AHI* apnea hypopnea index, *ODI* oxygen desaturation index, *ESS* the Epworth sleeping scale, *PTCA* percutaneous transluminal coronary angioplasty, *CABG* coronary artery bypass graft, *TIA* transient ischemic attack, *ARB* angiotensin receptor inhibitors, *ACE* angiotensin converting enzyme, *CCB* calcium channel blocker

### The impact of medication on survival and secondary endpoints

Chi-square test was used to estimate the relationship between medications and the endpoints (Table 2). Clopidogrel, angiotensin-converting enzyme inhibitors (ACE inhibitors), and nitrates were related to increase in mortality in participants with comorbid OSA and CV/CeV.

**Table 2:** The effect of medications on survival and secondary endpoints based on Chi-square tests

Medications	Death		Secondary endpoints	
	Chi-square	*P*	Chi-square	*P*
Aspirin	0.084	0.771	0.205	0.651
Clopidogrel	5.738	0.017	1.125	0.289
Statin	2.024	0.155	0.580	0.446
Beta-blocker	0.237	0.627	0.033	0.855
ARB	2.257	0.133	1.974	0.160
ACE inhibitors	4.058	0.044	2.139	0.144
CCB	0.383	0.536	0.821	0.365
Nitrates	5.304	0.021	2.918	0.088

*ARB* angiotensin receptor inhibitors, *ACE* angiotensin converting enzyme, *CCB* calcium channel blocker

**Fig. 1:**
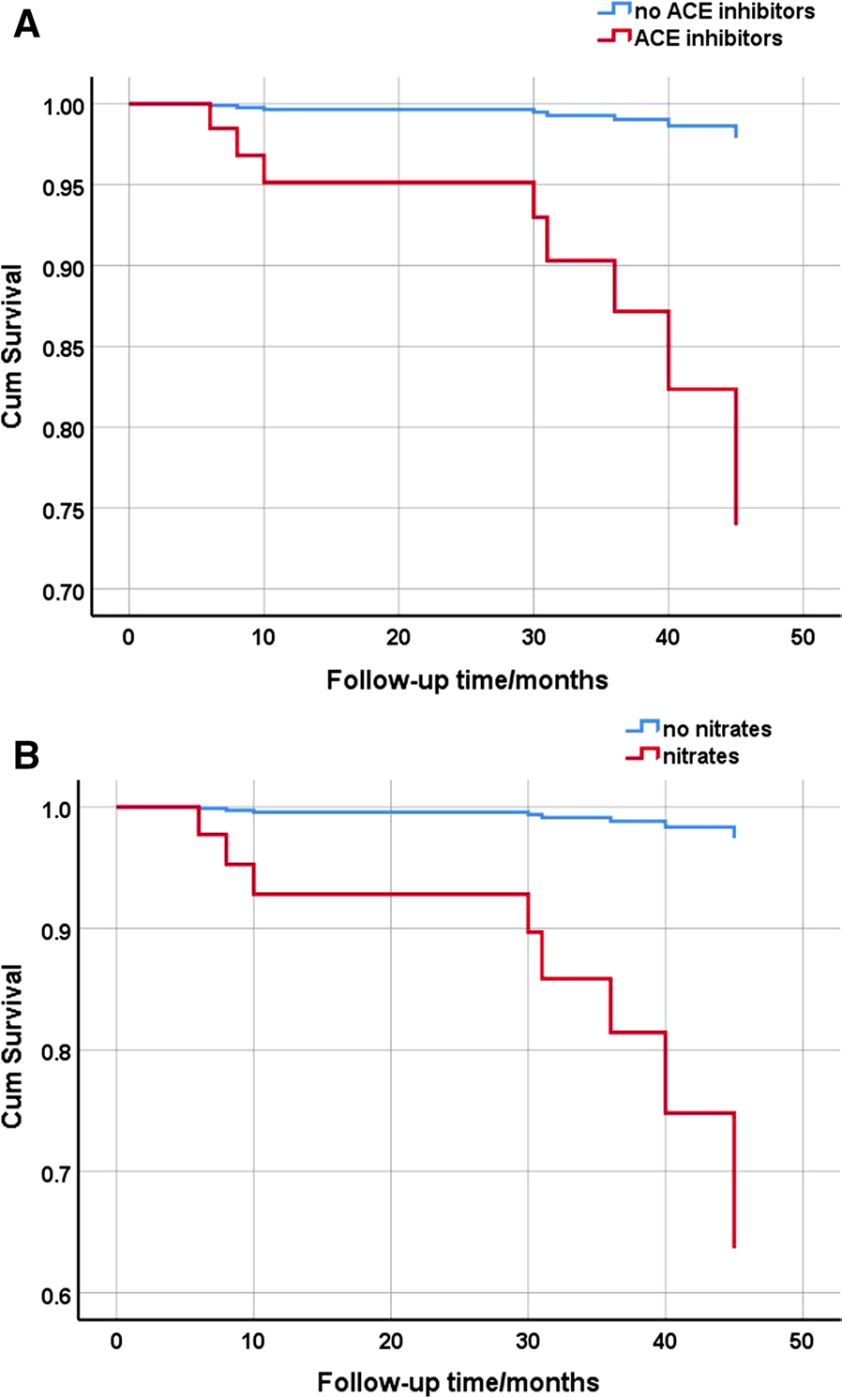
The effect of risk factors and medications on survival and total endpoints. **a** Survival curve of ACE inhibitors for patients with comorbidity OSA and cardiovascular or cerebrovascular diseases. **b** Survival curve of nitrates for patients with comorbidity OSA and cardiovascular or cerebrovascular diseases

Kaplan–Meier analysis was used to estimate the impact of sex, age, obesity, CPAP treatment, and medication on the primary and secondary endpoints (Table 3). The results suggested that clopidogrel, ACE inhibitors, and nitrates were associated with a reduction in survival. Sex, age, obesity, CPAP treatment, and medications were entered into Cox regression analysis to estimate the risk factors for the primary and secondary endpoints. The results suggested that ACE inhibitors and nitrates were independently associated with a reduction in survival (chi-square = 23.690, *P* = 0.022). The mortality rate of ACE inhibitors was 14.130 times that of no ACE inhibitors (*P* = 0.034, 95% CI 1.217–164.083) (Fig. 1a). The mortality rate of nitrate usage was 17.445 times that of no nitrate usage (*P* = 0.033, 95% CI 1.258–242.279) (Fig. 1b). Because the coefficients did not converge, no further Cox regression models were generated for the secondary endpoints.

**Table 3:** The effect of risk factors and medications on survival and secondary endpoints based on Kaplan–Meier analysis

	Death		Total endpoints	
	Chi-square	*P*	Chi-square	*P*
Female	0.096	0.756	0.145	0.703
Old age(age ≥ 65 years)	0.044	0.833	0.098	0.755
Obesity(BMI ≥ 25 kg/m^2^)	1.397	0.237	3.168	0.075
CPAP	0.299	0.585	0.771	0.380
Aspirin	0.115	0.735	0.088	0.766
Clopidogrel	6.425	0.011	1.65	0.199
Statin	2.161	0.142	0.994	0.319
Beta-blocker	0.176	0.675	0.083	0.773
ARB	2.355	0.125	2.341	0.126
ACE inhibitors	5.071	0.024	3.307	0.069
CCB	0.391	0.532	0.686	0.408
Nitrates	6.417	0.011	3.087	0.079

*CPAP* continues positive airway pressure, *BMI* body mass index, *ARB* angiotensin receptor inhibitors, *ACE* angiotensin converting enzyme, *CCB* calcium channel blocker

### The effect of medications on prognosis in CPAP group and usual care group

Chi-square test (Table 4) and Kaplan–Meier analysis were used to estimate the relationship between medications and endpoints in the CPAP and usual care groups, respectively.

**Table 4:** The effect of medications based on Chi-square tests in CPAP and usual care group

Medications	CPAP group				Usual care group			
	Death		Secondary endpoints		Death		Secondary endpoints	
	Chi-square	*P*	Chi-square	*P*	Chi-square	*P*	Chi-square	*P*
Aspirin	0.030	0.862	0.001	0.970	0.390	0.532	0.189	0.664
Clopidogrel	0.729	0.393	0.191	0.662	5.397	0.020	0.856	0.355
Statin	1.575	0.209	0.072	0.789	0.760	0.383	1.564	0.211
Beta-blocker	0.117	0.733	0.107	0.743	0.157	0.692	0.182	0.670
ARB	1.938	0.164	0.666	0.414	0.652	0.419	1.295	0.255
ACE inhibitors	7.058	0.008	4.033	0.045	0.215	0.643	0.065	0.799
CCB	3.252	0.071	0.002	0.962	0.452	0.501	1.648	0.199
Nitrates	0.103	0.748	0.300	0.584	6.520	0.011	4.059	0.044

*CPAP* continues positive airway pressure, *ARB* angiotensin receptor inhibitors, *ACE* angiotensin converting enzyme, *CCB* calcium channel blocker

Both chi-square test and Kaplan–Meier analysis suggested that ACE inhibitors were associated with increased mortality (Kaplan–Meier analysis: chi-square = 9.033, *P* = 0.003) and secondary endpoints (Kaplan–Meier analysis: chi-square = 4.134, *P* = 0.042) in the CPAP group. In participants with good CPAP adherence (CPAP usage ≥ 4 h per night), no significant risk factors were found.

Both chi-square tests and Kaplan–Meier analysis suggested that clopidogrel (chi-square = 5.312, *P* = 0.021) and nitrates (chi-square = 6.417, *P* = 0.011) were associated with reduced survival in the usual care group.

Sex, age, obesity, and medications were entered into Cox regression analysis to estimate the risk factors for the primary and secondary endpoints in both groups. Because the coefficients did not converge, no further Cox regression models were generated.

## Discussions

In this post hoc analysis of single-center data from the SAVE study, we found that usage of ACE inhibitors and nitrates were related to the decrease in survival in patients with comorbid OSA and cardiovascular/cerebrovascular disease; ACE inhibitors was related to the increase in mortality and secondary endpoints in the CPAP group, but not in patients with good CPAP adherence; Clopidogrel and nitrates were related with a decrease in survival in the usual care group, but not in the CPAP group. Interactions were observed between OSA and cardiovascular/cerebrovascular medications. The CPAP treatment may alleviate the negative effects of clopidogrel and nitrates on the survival of patients with comorbid OSA and CV/CeV. The interaction between OSA and medications, and CPAP and medications may conceal the positive effects of CPAP on the prognosis of patients with comorbid OSA and CV/CeV.

ACE inhibitors act by inhibiting the renin–angiotensin–aldosterone system. Captopril, ramipril, trandolapril, and enalapril have been reported to reduce morbidity and mortality in patients with left ventricular dysfunction or heart failure after myocardial infarction [6–9]. According to the 2019 European Society of Cardiology Guidelines for the management of chronic coronary syndromes, ACE inhibitors are recommended for the treatment of patients with chronic coronary syndromes comorbid with hypertension, left ventricular ejection fraction ≤ 40%, diabetes, or chronic kidney disease unless contraindicated [10]. In the Heart Outcomes Prevention Evaluation Study, ramipril significantly reduced the rates of death, myocardial infarction, and stroke in patients at high risk for cardiovascular events, but without left ventricular dysfunction or heart failure [11]. The effects of ACE inhibitors on stable coronary artery disease without heart failure are controversial. The European trial on Reduction Of cardiac events with Perindopril in patients with stable coronary Artery disease study indicated that among patients with stable coronary heart disease without heart failure, perindopril can improve outcomes [12]. However, the Pacing, graded Activity, and Cognitive behavior therapy; a randomized Evaluation study suggested that the addition of trandolapril provided no further benefit in terms of outcome in patients with stable coronary artery disease and normal or slightly reduced left ventricular function [13].

However, the relationship between OSA and ACE inhibitors remains largely unknown. Genetic studies have suggested that genetically reduced ACE activity may be a causal risk factor for OSA. There was a 32.4–37.4% increased risk of OSA by a reduction of 1 U/L in ACE activity [14]. However, the above findings were not conclusive, and the relationship between acquired ACE activity reduction by ACE inhibitors and OSA has not been thoroughly studied. Case reports have shown that the use of ACE inhibitors promotes OSA-like symptoms, such as nasal obstruction, snoring, daytime sleepiness, and witness sleep apnea [15], and reduces CPAP adherence [16] in patients with ACE inhibitor-related cough, but not in patients with good tolerance for ACE inhibitors.

In this study, ACE inhibitors were found to be independent risk factors for mortality. Moreover, ACE inhibitors were associated with increased mortality in the CPAP group but not in patients with good CPAP adherence. We speculate that there may be two reasons for this finding. First, according to the inclusion and exclusion criteria, the participants in this study had stable coronary artery disease with normal or slightly impaired left ventricular function and did not benefit from ACE inhibitors for conclusive evidence. Second, ACE inhibitors may interact with OSA, and CPAP may relieve the negative impact of OSA to some extent. Eighteen participants were administered ACE inhibitors. Perindopril was used in 11 participants (61.1%), benazepril in 3 (16.7%), ramipril in 3 (16.7%), and fosinopril in 1 (5.6%). Although perindopril has been reported to improve outcomes in patients with stable coronary heart disease without heart failure [12], the complicated interaction between ACE inhibitors and OSA might impair this effect. Further investigation is needed to determine the relationship and mechanism among OSA, CPAP, and ACE inhibitors.

Antiplatelet treatment has been recommended for both cardiovascular and cerebrovascular diseases [10, 17]. Clopidogrel should be taken for secondary prevention in patients with stroke or TIA [17]. Increased platelet reactivity was noted in patients with OSA. Platelet reactivity was higher in OSA patients who underwent percutaneous coronary intervention despite receiving a loading dose of clopidogrel, and OSA was an independent predictor of high platelet reactivity to clopidogrel [18]. Patients with OSA are more likely to have high residual on-treatment platelet reactivity after clopidogrel therapy [19]. In this study, clopidogrel was associated with increased mortality in the overall study population and the usual care group but not in the CPAP group. The increase in platelet reactivity in patients with OSA may require dose re-titration of clopidogrel or other medications. Whether CPAP treatment could decrease platelet over-reactivity in patients with OSA requires further investigation.

The relationship between nitrate and OSA remains unclear. Circulating nitric oxide is suppressed in OSA, and this effect is reversible with CPAP treatment [20]. However, OSA does not affect endothelium-independent nitroglycerin-induced dilation of the brachial artery [21, 22]. In this study, nitrate was found to be an independent factor for mortality in the overall study population. Short-acting nitrates provide immediate relief from effort-related angina. Long-acting nitrates are a second-line option when initial treatment with a beta-blocker and/or dihydropyridine calcium channel blockers is contraindicated, poorly tolerated, and inadequate in controlling angina symptoms [10]. Patients taking long-acting nitrates may have a worse underlying disease state, and hence a worse prognosis.

This study has some limitations. First, participants were assigned to different groups using CPAP instead of a specific medication. Second, for most participants, many medications were taken at the same time, and the interaction among different medications could hardly be estimated. Third, the sample size was relatively small, and comorbidities and medications may become important confounders even in the adjusted multivariate analysis. For these reasons, despite the significant association between some medications and poor prognosis, we cannot infer a cause-effect relationship.

## Conclusions

Overall, OSA may predispose patients with CV/CeV and CV/CeV medications to a negative effect. CPAP treatment may neutralize some of the negative effects caused by OSA by relieving chronic intermittent hypoxia. Further research is needed to determine the relationship among OSA, CPAP, and cardiovascular/cerebrovascular medications.

## Data Availability

The datasets used and/or analyzed during the current study are available from the corresponding author upon reasonable request.

## Abbreviations

OSA: Obstructive sleep apnea
CV: Cardiovascular disease
CeV: Cerebrovascular disease
TIA: Transient ischemic attack
MACCEs: Major adverse cardiac and cerebrovascular events
SAVE: The Sleep Apnea Cardiovascular Endpoints
CPAP: Continuous positive airway pressure
ACE inhibitors: Angiotensin-converting enzyme inhibitors
ARB: Angiotensin receptor blocker
CCB: Calcium channel blocker
